# Recognition of a Sausage-Shaped Pancreas on Non-contrast Computed Tomography as an Early Clue to Type 2 Autoimmune Pancreatitis in a Young Adult

**DOI:** 10.7759/cureus.110250

**Published:** 2026-06-04

**Authors:** Shamas Rafique, Iqra Rafiq

**Affiliations:** 1 Medicine and Surgery, Xinjiang Medical University, Urumqi, CHN; 2 Internal Medicine, BayCare Medical PC, New York, USA; 3 Medicine and Surgery, BeeWell International Hospital, Islamabad, PAK; 4 Internal Medicine, First Affiliated Hospital of Xinjiang Medical University, Urumqi, CHN; 5 General Medicine, Services Hospital Lahore, Lahore, PAK; 6 Medicine and Surgery, First Affiliated Hospital of Xinjiang Medical University, Urumqi, CHN; 7 Family Medicine, Family Medical Health Care PLLC, New York, USA

**Keywords:** autoimmune pancreatitis, granulocytic epithelial lesions, idiopathic duct‑centric pancreatitis, non‑contrast computed tomography, sausage‑shaped pancreas, type 2 autoimmune pancreatitis, young adult

## Abstract

Autoimmune pancreatitis (AIP) is an uncommon form of chronic pancreatitis characterized by immune-mediated inflammation and a dramatic response to corticosteroid therapy. Two distinct subtypes are recognized, each with differing clinical, serological, and histopathologic profiles. Type 2 AIP predominantly affects younger patients and is not associated with elevated serum immunoglobulin G4 levels, making the diagnosis particularly challenging. We report a 28‑year‑old man who presented with recurrent epigastric pain and elevated serum lipase. Non‑contrast computed tomography (CT) revealed no peripancreatic fat stranding or fluid collection; however, subtle loss of the normal pancreatic lobular architecture produced a sausage‑shaped configuration of the pancreas. Further evaluation with magnetic resonance cholangiopancreatography demonstrated diffuse irregular narrowing of the main pancreatic duct. Endoscopic ultrasound‑guided biopsy confirmed idiopathic duct‑centric pancreatitis with granulocytic epithelial lesions, consistent with type 2 AIP. Complete clinical, biochemical, and radiologic resolution was achieved following corticosteroid therapy. This case highlights that a sausage‑shaped pancreas on non‑contrast CT, even in the absence of typical inflammatory features, should prompt consideration of AIP in young adults.

## Introduction

Autoimmune pancreatitis (AIP) is a distinct form of chronic pancreatitis that is immune-mediated and highly responsive to corticosteroid treatment [1]. The International Consensus Diagnostic Criteria (ICDC), published in 2011, classified AIP into two distinct subtypes: type 1 AIP, also known as lymphoplasmacytic sclerosing pancreatitis, which represents the pancreatic manifestation of immunoglobulin G4 (IgG4)‑related disease; and type 2 AIP, referred to as idiopathic duct‑centric pancreatitis (IDCP), which is characterized by granulocytic epithelial lesions (GELs) and is typically confined to the pancreas [1,2].

The two subtypes display markedly different epidemiologic profiles. Type 1 AIP predominantly affects elderly males, with a mean age at diagnosis exceeding 60 years, whereas type 2 AIP affects younger individuals, with a mean age of approximately 40 years and a relatively equal sex distribution [3]. Although type 2 AIP is reported more frequently in Europe and North America, it remains an uncommon diagnosis worldwide [4,5].

The classic imaging finding on computed tomography (CT) is a diffusely enlarged, sausage‑shaped pancreas with loss of the normal lobular contours [6]. On magnetic resonance cholangiopancreatography (MRCP), diffuse irregular narrowing of the main pancreatic duct is a characteristic feature [7]. Type 2 AIP poses a particular diagnostic challenge because serum IgG4 levels are typically normal [8,9]. Consequently, histopathologic confirmation via endoscopic ultrasound (EUS)-guided biopsy is often required [2,10]. GELs represent the defining histologic feature of type 2 AIP [2,10].

Treatment with oral corticosteroids remains first‑line therapy, with remission rates exceeding 90% [11]. Relapse rates are significantly higher in type 1 AIP compared with type 2 disease [3,11].

We present a case of type 2 AIP in a 28‑year‑old man whose diagnosis was prompted by the subtle finding of a sausage‑shaped pancreas on non‑contrast CT, underscoring the importance of recognizing this morphologic sign in young patients presenting with unexplained pancreatitis.

## Case presentation

A 28‑year‑old Hispanic man with no significant past medical or surgical history presented to the emergency department with a two‑week history of intermittent, dull epigastric pain radiating to the back. He denied alcohol consumption, tobacco use, or illicit drug use. He also denied recent trauma, medication changes, constitutional symptoms, or a family history of pancreatic disease. Physical examination revealed mild epigastric tenderness to deep palpation without rebound tenderness, guarding, abdominal distension, or palpable masses. Vital signs were within normal limits.

Initial laboratory evaluation was performed to assess acute pancreatitis, hepatobiliary pathology, metabolic etiologies, and autoimmune disease. The most significant abnormality was a markedly elevated serum lipase level (more than six times the upper limit of normal), which, together with the characteristic epigastric pain, fulfilled the Atlanta criteria for acute pancreatitis. Serum amylase was normal. Mild elevation of inflammatory markers was present, while liver function tests, serum calcium, and triglyceride levels were within normal limits, making gallstone disease and metabolic pancreatitis less likely. A complete blood count demonstrated no leukocytosis or eosinophilia. Serum IgG4 testing and autoimmune serologies were obtained to evaluate for AIP and associated immune-mediated conditions. The admission laboratory findings are summarized in Table 1.

**Table 1:** Admission laboratory evaluation. AST: aspartate aminotransferase; ALT: alanine aminotransferase; ALP: alkaline phosphatase; IgG4: immunoglobulin G4; IgG: immunoglobulin G; ANA: antinuclear antibody; ASMA: anti‑smooth muscle antibody; CRP: C‑reactive protein.

Parameter	Patient value	Reference range
Serum Lipase (U/L)	384	0-60
Serum Amylase (U/L)	62	30-110
AST (U/L)	28	10-40
ALT (U/L)	34	10-50
ALP (U/L)	78	40-130
Total Bilirubin (mg/dL)	0.7	0.1-1.2
Direct Bilirubin (mg/dL)	0.2	0.0-0.3
Serum Calcium (mg/dL)	9.4	8.5-10.5
Serum Triglycerides (mg/dL)	112	<150
Serum IgG4 (mg/dL)	88	4-135
Total IgG (mg/dL)	1250	700-1600
ANA	Negative	Negative
ASMA	Negative	Negative
CRP (mg/L)	18.4	<5.0

Radiologic evaluation was initiated with a non‑contrast CT scan of the abdomen, performed as part of the emergency department’s acute abdomen protocol for a young patient with elevated lipase but no contraindication to contrast. The non‑contrast study was chosen to rapidly screen for alternative surgical pathology while avoiding contrast exposure and minimizing cumulative radiation by bypassing a multi‑phasic pancreatic protocol. Although no findings of acute interstitial pancreatitis (such as peripancreatic fat stranding or fluid) were identified, careful morphologic assessment of the pancreas demonstrated subtle diffuse abnormalities suggestive of AIP.

The liver, spleen, kidneys, and adrenal glands appeared normal. There was no peripancreatic fat stranding, fluid collection, pancreatic necrosis, or focal pancreatic mass. However, close inspection revealed diffuse loss of the normal pancreatic lobular architecture with obliteration of the pancreatic clefts, imparting a smooth, homogeneous, sausage-shaped morphology to the entire gland (Figure 1). No biliary ductal dilatation, lymphadenopathy, or ascites was identified.

**Figure 1:**
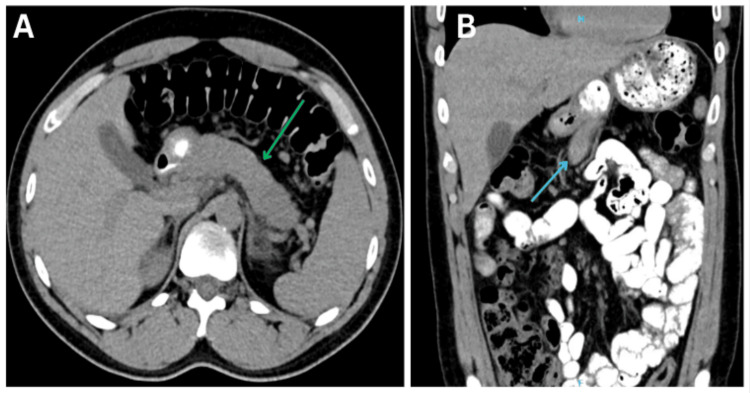
Non-contrast CT images demonstrating diffuse sausage-shaped morphology of the pancreas at initial presentation. (A) Axial non-contrast CT image at the level of the pancreatic body and tail demonstrates diffuse loss of the normal lobulated pancreatic contour with obliteration of the pancreatic clefts, resulting in smooth sausage-shaped enlargement of the gland (green arrow). No peripancreatic fat stranding, fluid collection, or pancreatic necrosis is identified. (B) Coronal reconstructed image confirms diffuse smooth enlargement of the pancreas with effacement of the normal lobular architecture (blue arrow).

Given the discordance between the markedly elevated serum lipase and the paucity of inflammatory changes on CT, additional evaluation with MRCP was performed; MRCP demonstrated diffuse, irregular narrowing of the main pancreatic duct from the head to the tail without upstream ductal dilatation, a characteristic feature of AIP. Alternative causes of pancreatitis were systematically excluded: serum triglyceride and calcium levels were normal, and there was no history of alcohol use, medication exposure, abdominal trauma, or biliary disease. Autoimmune serologies remained negative. EUS was subsequently pursued and demonstrated a diffusely hypoechoic pancreas with loss of the normal lobulated echotexture; EUS‑guided fine‑needle biopsy of the pancreatic body was performed for histopathologic confirmation. (Note: MRCP and EUS images are not available for this report; only pre‑ and post‑treatment CT images were retained for publication.)

Fine‑needle biopsy specimens underwent histopathologic and immunohistochemical evaluation (Table 2).

**Table 2:** Histopathologic and immunohistochemical findings from EUS‑guided pancreatic biopsy EUS: endoscopic ultrasound; AIP: autoimmune pancreatitis; IgG4: immunoglobulin G4.

Histopathologic feature	Findings	Diagnostic significance
Pancreatic duct epithelium	Neutrophilic infiltration with granulocytic epithelial lesions	Characteristic feature of type 2 AIP
Periductal inflammatory infiltrate	Mild lymphoplasmacytic inflammation	Supports immune-mediated pancreatic injury
Fibrosis	Focal periductal fibrosis without storiform pattern	Favors type 2 AIP over type 1 disease
Obliterative phlebitis	Not identified	Absence argues against type 1 AIP
IgG4‑positive plasma cells	Rare/scant positive cells	Inconsistent with IgG4‑related disease
Overall interpretation	Idiopathic duct‑centric pancreatitis	Consistent with type 2 AIP

Histopathologic examination demonstrated idiopathic duct‑centric pancreatitis with neutrophilic infiltration of the ductal epithelium, forming GELs. Additional findings included periductal lymphoplasmacytic infiltration and fibrosis without storiform fibrosis or obliterative phlebitis. Immunohistochemical analysis demonstrated only scant IgG4‑positive plasma cells, findings consistent with type 2 AIP rather than type 1 disease.

A definitive diagnosis of type 2 AIP was established based on level 1 imaging findings (diffuse pancreatic enlargement with loss of lobulation and diffuse irregular narrowing of the main pancreatic duct) and level 1 histology (idiopathic duct‑centric pancreatitis with GELs), according to the ICDC, based on the combined CT morphology and the clinically verified MRCP ductal findings.

The patient was started on oral prednisone 40 mg/day. Within one week, his epigastric pain completely resolved, and repeat laboratory studies demonstrated normalization of serum lipase and C‑reactive protein, confirming biochemical remission (Table 3). After four weeks, prednisone was gradually tapered by 5 mg every two weeks. Follow‑up non‑contrast CT performed three months later demonstrated restoration of the normal pancreatic lobular architecture with reappearance of the pancreatic clefts and complete resolution of the sausage‑shaped morphology (Figure 2). Follow-up MRCP at four months confirmed interval resolution of the diffuse pancreatic ductal narrowing.

**Table 3:** Follow‑up laboratory values after one week of corticosteroid therapy. CRP: C-reactive protein; IgG4: immunoglobulin G4; U/L: units per liter; mg/L: milligrams per liter; mg/dL: milligrams per deciliter.

Parameter	Patient value	Reference range
Serum Lipase (U/L)	42	0-60
Serum Amylase (U/L)	58	30-110
CRP (mg/L)	2.1	<5.0
Serum IgG4 (mg/dL)	90	4-135

**Figure 2:**
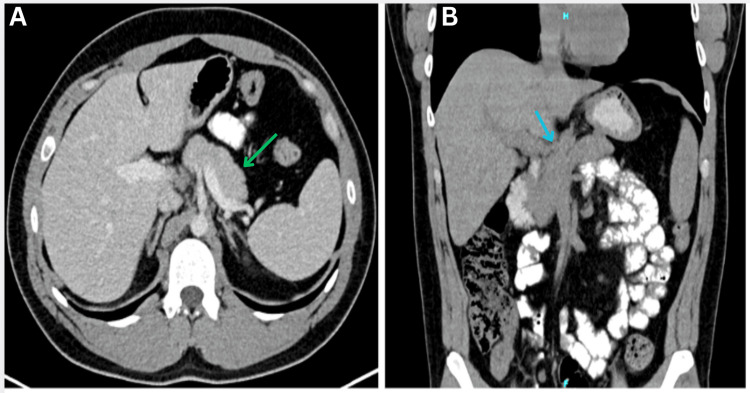
Follow-up non-contrast CT after corticosteroid therapy. (A) Axial follow‑up non‑contrast CT obtained three months after initiation of prednisone demonstrates restoration of the normal pancreatic lobulated contour with reappearance of pancreatic clefts and complete resolution of the previously observed sausage‑shaped morphology (green arrow). (B) A coronal reconstructed image confirms normalization of the pancreatic contour and restoration of the normal lobulated architecture without residual diffuse enlargement (blue arrow), consistent with radiologic remission of autoimmune pancreatitis.

At six‑month follow‑up, the patient remained asymptomatic on a prednisone dose of 5 mg daily, which represented the final step of a prolonged taper with planned discontinuation over the subsequent weeks, given the low relapse risk in type 2 AIP. There was no clinical, biochemical, or radiologic evidence of disease relapse.

## Discussion

Type 2 AIP remains a diagnostically challenging entity because of its rarity, younger age at presentation, absence of elevated serum IgG4 levels, and frequent overlap with other causes of pancreatitis. This case highlights an uncommon presentation of type 2 AIP in a 28‑year‑old man in whom the diagnosis was initially suggested by a subtle morphologic abnormality on non‑contrast CT.

The ICDC, published in 2011, established a standardized framework for diagnosing AIP using five cardinal features: pancreatic parenchymal imaging, ductal imaging, serology, other organ involvement, pancreatic histology, and response to corticosteroid therapy [1]. In the present case, a definitive diagnosis was established based on level 1 imaging findings and level 1 histopathology, demonstrating idiopathic duct‑centric pancreatitis with GELs.

Type 1 AIP has a well-established demographic profile, with a mean age at diagnosis exceeding 60 years and a strong male predominance, whereas type 2 AIP affects younger patients, with a mean age in the fifth decade and no clear sex predilection [3,12]. The patient’s age of 28 years makes this presentation distinctly uncommon. Although type 2 AIP has been reported in adolescents and young adults, most large international cohorts report a substantially older mean age at diagnosis [3,13].

The classic radiologic hallmark of diffuse AIP is a sausage‑shaped pancreas with diffuse loss of the normal lobulated contour [6]. Additional findings on contrast-enhanced CT may include delayed homogeneous enhancement and a low-attenuation capsule-like rim [6,7]. Although contrast‑enhanced pancreatic protocol CT remains the preferred imaging modality for evaluation of suspected AIP, this case demonstrates that careful assessment of pancreatic morphology on non‑contrast imaging may still provide important diagnostic clues. In this patient, diffuse effacement of pancreatic lobulations and obliteration of pancreatic clefts produced the characteristic smooth sausage‑shaped morphology despite the absence of overt inflammatory changes such as peripancreatic fat stranding, fluid collection, or necrosis. Once the characteristic sausage‑shaped morphology was identified on non‑contrast CT and corroborated by MRCP and EUS findings, a repeat contrast‑enhanced CT was deferred to avoid additional radiation and iodinated contrast exposure; the combination of non‑contrast CT morphology, MRCP ductal assessment, and EUS‑guided biopsy was considered sufficient for diagnostic decision‑making.

MRCP further strengthened the diagnostic impression by demonstrating diffuse irregular narrowing of the main pancreatic duct without upstream ductal dilatation, a characteristic ductal manifestation of AIP [7]. The combination of diffuse glandular enlargement and long‑segment ductal narrowing is highly suggestive of AIP and helps distinguish it from pancreatic adenocarcinoma, which more commonly produces abrupt focal ductal obstruction with upstream ductal dilatation. The differential diagnosis for a diffusely enlarged, sausage-shaped pancreas also includes groove pancreatitis, pancreatic lymphoma, and infiltrating ductal adenocarcinoma. In this case, the absence of cystic duodenal wall thickening argued against groove pancreatitis; the lack of associated lymphadenopathy and the histopathologic absence of atypical lymphoid cells excluded lymphoma; and the long‑segment, smooth ductal narrowing without an abrupt cutoff or upstream dilatation, together with the biopsy findings, effectively ruled out adenocarcinoma.

Serologic evaluation has limited utility in type 2 AIP because serum IgG4 levels are frequently normal [8,14]. Although elevated IgG4 levels demonstrate high specificity for type 1 disease, normal values do not exclude AIP, particularly in younger patients [14,15]. Consequently, histopathologic confirmation becomes especially important when clinical suspicion persists despite negative serologic findings.

Histopathologically, type 2 AIP is characterized by GELs consisting of neutrophilic infiltration of the pancreatic ductal epithelium [2,10]. These lesions represent the defining histologic feature of idiopathic duct‑centric pancreatitis and allow distinction from type 1 disease, which is characterized by dense IgG4‑positive lymphoplasmacytic infiltration, storiform fibrosis, and obliterative phlebitis [2]. In the present case, EUS‑guided biopsy demonstrated classic GELs with only scant IgG4‑positive plasma cells, confirming the diagnosis of type 2 AIP.

Corticosteroids remain first‑line therapy for both subtypes of AIP, with remission rates exceeding 90% [11]. Clinical and biochemical responses are typically rapid and dramatic, and the therapeutic response itself serves as a supportive diagnostic criterion within the ICDC framework [1,11]. In this patient, prompt resolution of symptoms, normalization of serum lipase and inflammatory markers, and restoration of normal pancreatic morphology after corticosteroid therapy provided additional confirmation of the diagnosis. The decision to continue a low dose of prednisone (5 mg daily) at six months reflected a slow final taper rather than long-term maintenance, consistent with the known low relapse risk of type 2 AIP; the dose was scheduled for discontinuation within weeks.

This report has several limitations. As a single‑case presentation, the findings may not be generalizable. Furthermore, the original MRCP and EUS images are not available for this retrospective report; however, the clinical documentation of diffuse pancreatic duct narrowing and GELs is sufficient to meet the ICDC for definitive type 2 AIP. Longer‑term follow‑up remains necessary to monitor for disease recurrence and for the potential development of exocrine or endocrine pancreatic insufficiency. The patient has been scheduled for annual surveillance comprising clinical evaluation, serum lipase and IgG4 measurement, and cross-sectional imaging to monitor for disease relapse and for the development of exocrine or endocrine insufficiency.

Recognition of subtle diffuse pancreatic contour abnormalities on non-contrast CT may allow earlier identification of AIP before the development of overt inflammatory changes. In younger patients presenting with unexplained pancreatitis and normal serum IgG4 levels, diffuse sausage‑shaped pancreatic morphology should prompt further evaluation with MRCP and EUS‑guided tissue sampling.

This case emphasizes several important clinical points. First, AIP should remain within the differential diagnosis of unexplained pancreatitis even in patients younger than 30 years of age. Second, subtle morphologic abnormalities on non‑contrast CT may represent the earliest radiologic manifestation of disease. Third, normal serum IgG4 levels do not exclude AIP, particularly type 2 disease. Early recognition is important because delayed diagnosis may result in recurrent pancreatitis, chronic pancreatic injury, and unnecessary invasive procedures or surgical intervention.

## Conclusions

This case demonstrates that a sausage‑shaped pancreas on non‑contrast CT, reflecting diffuse loss of the normal pancreatic lobular architecture, may represent the earliest and only imaging manifestation of AIP. Recognition of this subtle morphologic finding is critical, particularly in young adults presenting with unexplained pancreatitis and normal serum IgG4 levels. Comprehensive evaluation using the ICDC facilitates accurate diagnosis and subtype classification while helping exclude pancreatic malignancy and avoid unnecessary surgical intervention. Type 2 AIP should therefore be included in the differential diagnosis of pancreatitis in patients younger than 30 years of age.

## References

[1] Shimosegawa T, Chari ST, Frulloni L (2011). International Consensus Diagnostic Criteria for autoimmune pancreatitis: guidelines of the International Association of Pancreatology. Pancreas.

[2] Zhang L, Chari S, Smyrk TC (2011). Autoimmune pancreatitis (AIP) type 1 and type 2: an international consensus study on histopathologic diagnostic criteria. Pancreas.

[3] Kamisawa T, Chari ST, Giday SA (2011). Clinical profile of autoimmune pancreatitis and its histological subtypes: an international multicenter survey. Pancreas.

[4] Hart PA, Zen Y, Chari ST (2015). Recent advances in autoimmune pancreatitis. Gastroenterology.

[5] Hart PA, Kamisawa T, Brugge WR (2013). Long-term outcomes of autoimmune pancreatitis: a multicentre, international analysis. Gut.

[6] Crosara S, D'Onofrio M, De Robertis R, Demozzi E, Canestrini S, Zamboni G, Pozzi Mucelli R (2014). Autoimmune pancreatitis: multimodality non-invasive imaging diagnosis. World J Gastroenterol.

[7] Manfredi R, Frulloni L, Mantovani W, Bonatti M, Graziani R, Pozzi Mucelli R (2011). Autoimmune pancreatitis: pancreatic and extrapancreatic MR imaging-MR cholangiopancreatography findings at diagnosis, after steroid therapy, and at recurrence. Radiology.

[8] Sah RP, Chari ST, Pannala R (2010). Differences in clinical profile and relapse rate of type 1 versus type 2 autoimmune pancreatitis. Gastroenterology.

[9] Kamisawa T, Okamoto A (2008). IgG4-related sclerosing disease. World J Gastroenterol.

[10] Deshpande V, Gupta R, Sainani N (2011). Subclassification of autoimmune pancreatitis: a histologic classification with clinical significance. Am J Surg Pathol.

[11] Pădureanu V, Drăgoescu AN, Pădureanu R, Rośu MM, Rădulescu D, Dop D, For Ofoiu MC (2024). Treatment approaches in autoimmune pancreatitis (Review). Biomed Rep.

[12] Chari ST, Kloeppel G, Zhang L, Notohara K, Lerch MM, Shimosegawa T (2010). Histopathologic and clinical subtypes of autoimmune pancreatitis: the Honolulu consensus document. Pancreas.

[13] Scheers I, Palermo JJ, Freedman S (2017). Autoimmune pancreatitis in children: characteristic features, diagnosis, and management. Am J Gastroenterol.

[14] Lee SC, Yang CH, Chang CT, Yu KH (2022). Diagnostic utility of serum IgG4 in autoimmune pancreatitis: an updated comprehensive systematic review and meta-analysis. J Clin Gastroenterol.

[15] Ghazale A, Chari ST, Zhang L (2008). Immunoglobulin G4-associated cholangitis: clinical profile and response to therapy. Gastroenterology.

